# Editorial: Global health and warfare: assessing the broad impacts of conflict on public health

**DOI:** 10.3389/fpubh.2025.1690317

**Published:** 2025-09-29

**Authors:** Stefano Orlando, Paolo Vineis, Pirous Fateh-Moghadam

**Affiliations:** ^1^Department of Biomedicine and Prevention, Università degli Studi di Roma Tor Vergata, Roma, Italy; ^2^School of Public Health, Imperial College London, London, United Kingdom; ^3^Department of Prevention, Local Health Unit, Trento, Italy

**Keywords:** armed conflict, refugees and IDPs, maternal and child health (MCH), mental health, immunization, human rights, humanitarian health response, global health

## 1 Introduction

Armed conflict remains one of the most significant determinants of population health globally, producing wide-ranging, profound, and enduring impacts that extend far beyond the immediate battlefield. While combat operations directly affect military personnel, civilian populations increasingly bear the brunt of warfare. According to the World Health Organization, ~90% of conflict-related casualties since World War II have been among civilians, highlighting the disproportionate toll that modern conflicts exact on non-combatants (1). Furthermore, conflicts precipitate extensive indirect health consequences, which often surpass the immediate effects of violence itself. For instance, disrupted health systems, damaged infrastructure, displacement, and restricted access to healthcare services exacerbate mortality and morbidity far beyond direct injuries (2). A systematic analysis in The Lancet found that conflicts between 1990 and 2017 were associated with an estimated 29 million excess deaths due to indirect effects, including communicable diseases, malnutrition, and inadequate maternal and child healthcare services (3). These indirect effects persist long after conflicts end, shaping health outcomes for generations and posing significant challenges for global public health.

The large toll among civilians is the consequence of two characteristics of contemporary war, consistent with the nature of contemporary capitalism: (1) the role of sophisticated technologies, that allow devastating damage from remote locations, with much lower direct exposure of soldiers to the conflict theater (for example through the use of drones); (2) the market economy of weapons, which have become a commodity available to any militia, political group, tribe or ethnic minority (not to mention private citizens like in the United States). Concerning the first aspect, the fact that war is often made with the intermediation of technological devices like drones leads to reduced awareness of individual responsibility of soldiers and officers. The First World War (like many others) has seen a flourishing of literature on moral conflicts of soldiers and officers involved in war operations (think of Erich Maria Remarque or Emilio Lussu), an experience that does not seem to be repeated in current wars. Concerning the second aspect, the United States dominates the export of weapons with a 43% share in 2020–24, followed by France (9.6%), Russia (7.8%), China (5.9%), Germany (5.6%), Italy (4.8%), the United Kingdom (3.6%) and Israel (3.2%) (4). This ranking does not reflect the size of the countries, being disproportionally larger for the US and Israel than for other countries in relation to the population size; neither it reflects the size of other exports. Weapons are undoubtedly an important business, that flourished further after the Russian invasion of Ukraine; and are a commodity like others. The latter aspect goes together with a reduced sense of responsibility, both the moral awareness in the production and sale of weapons (business like others, within the global market) and the perception of moral implications in the use of weapons. This creates a situation not radically different from other planetary challenges today, like the impact of climate change and loss of biodiversity on the Global South, that is created by economic activities in high-income countries. Or even pandemics, whose origin tends to be distal from the affected populations, attenuating the sense of responsibility and making proximal measures less effective.

In recent years, the global landscape has witnessed a marked escalation in armed conflicts, leading to devastating humanitarian crises. The Russian invasion of Ukraine in 2022 stands as a significant catalyst, not only resulting in substantial casualties and displacement within Ukraine but also influencing global geopolitical tensions. This event has emboldened other geopolitical actors, contributing to a surge in conflicts worldwide. In the Gaza Strip, the conflict has reached unprecedented levels of lethality. The ongoing conflict in the Gaza Strip has inflicted severe harm on civilian populations. While various sources have reported casualty figures, the United Nations Office for the Coordination of Humanitarian Affairs (OCHA) emphasizes that these numbers are pending independent verification (5). In the meanwhile OCHA issues “Reported impact snapshots” specifying the source in case data are yet-to-be verified. At the time of writing the latest available data from the Gaza MoH included in the OCHA report are 61,158 fatalities and 151,442 injuries (as of the 6^th^ August 2025) (6). Several studies have been conducted to evaluate the accuracy of the Gaza MoH data (7–10) and no data fabrication could be found while one of the more recent studies, a capture-recapture analyses using different data sources, resulted in higher mortality figures compared to Gaza MoH data (10). As Colombo, who also contributed to this Research Topic, states in an editorial summarizing the results of the different estimates: “regardless of the true number of deaths, the suffering of Gazans has been immense. And it is not yet over” (11). The judges of the International Criminal Court (ICC) have found that there are reasonable grounds to believe that Israeli Prime Minister Benjamin Netanyahu, and former Israeli Minister of Defense Yoav Gallant “have committed the war crime of using starvation as a method of warfare and crimes against humanity of murder, persecution, and other inhumane acts, as a direct perpetrator, acting jointly with others. The Chamber also found reasonable grounds to believe that they are each responsible for the war crime of intentionally directing attacks against civilians as a superior” (12). Therefore the ICC issued arrest warrants on their behalf in addition to the arrest warrant against Mohammed Diab Ibrahim Al-Masri, more commonly known as Deif, Commander-in-Chief of the military wing of Hamas, accused of “crimes against humanity of murder, extermination, torture, and rape and other forms of sexual violence”. Furthermore the International Court of Justice is investigating the application of the convention on the prevention and punishment of the crime of genocide in the Gaza strip, and issued several provisional preventive measures in January 2024, reaffirmed in March 2024, to be taken by Israel in conformity with its obligations under the genocide convention, for example “to take all necessary and effective measures to ensure, without delay, in full co-operation with the United Nations, the unhindered provision at scale by all concerned of urgently needed basic services and humanitarian assistance, including food, water, electricity, fuel, shelter, clothing, hygiene and sanitation requirements, as well as medical supplies and medical care to Palestinians throughout Gaza (...)” (13). Israel notoriously ignored the prescriptions and the humanitarian situation in Gaza now is worse than ever giving even more reasons to believe in the appropriateness of applying the term genocidal to the military conduct of Israel. As a matter of fact, the Joint Public Health Statement on Gaza, issued on the 18th of July 2025 by the European Public Health Alliance (EPHA), The European Public Health Association (EUPHA), and the World Federation of Public Health Associations (WFPHA), recognizes the health crisis in Gaza as genocide-related and calls for urgent international action to address its devastating public health consequences (14). This position is consistent with statements from two Israeli human rights organizations, B'Tselem and Physicians for Human Rights Israel, which have also accused their own state of committing genocide (15, 16).

Since April 2023, Sudan has been embroiled in a devastating civil war between the Sudanese Armed Forces (SAF) and the Rapid Support Forces (RSF), resulting in significant civilian casualties. Estimates of civilian deaths vary widely, ranging from 20,000 to 150,000, highlighting the challenges in obtaining accurate data amid ongoing conflict. This uncertainty underscores the urgent need for comprehensive and verified assessments to fully comprehend the conflict's impact on the civilian population (17). These conflicts exemplify a broader trend of increasing global violence. The Armed Conflict Location and Event Data Project (ACLED) reports that, in the past 5 years, conflict levels have nearly doubled. In 2020, 104,371 conflict events were recorded; by 2025, this number approached 200,000, indicating a significant escalation in global instability (18).

## 2 This Research Topic

This Research Topic was launched to mobilize the public health community toward addressing the profound and widespread impacts of warfare on population health. The initiative aims to foster a deeper understanding of the health consequences of armed conflict, promote evidence-based interventions, inform policy-making, and strengthen international strategies aimed at conflict prevention, health system resilience, and humanitarian response.

We have included 21 papers in this Research Topic, representing a substantial scholarly contribution to the field. These papers explore critical themes, including: 1. Disruption of Healthcare and Infrastructure, 2. Forced Displacement and Refugee Health, 3. Disease Burdens, 4. Maternal and Reproductive Health, 5. Mental Health and Psychosocial Outcomes, and 6. Human Rights Violations and Civilian Casualties. Together, these studies provide comprehensive insights into the extensive health impacts associated with contemporary conflicts. From an overarching perspective, Levy provides a sweeping overview of conflict's direct (explosive weapon casualties) and indirect (malnutrition, mental health, displacement) impact, emphasizing that war always involves widespread human rights infractions.

### 2.1 Disruption of healthcare and infrastructure

Conflict frequently leads to insufficient staffing, scarce medical supplies, and infrastructural damage. For instance, a detailed case study in the Gaza Strip by Alamrain et al. revealed how the Orthopedic Department at Al-Aqsa Martyrs' Hospital was overwhelmed by trauma patients and forced to repurpose wards, create field dressing tents, and rely on external NGOs. A parallel situation emerges from the Tigray conflict: Gebru et al. found that war overshadowed COVID-19 prevention, crippling established emergency operation centers and contributing to a sharp spike in positivity rates once services partially resumed.

Additional complexity arises for specialized fields. In an investigation of pediatric urological procedures under volunteer campaigns, Aboalsamh et al. highlight how surgeons adopt creative solutions—telemedicine, multi-shift rotations, and reliance on local paraprofessionals—when resources are sparse. In Ukraine, Jonak et al. documented war-related ocular trauma among military personnel and civilians, reporting a high prevalence of macular and retinal injuries. Both studies highlight the stress placed on specialized departments when overall healthcare systems are compromised.

### 2.2 Forced displacement and refugee health

Adam et al. studied community-based mortality in Banadir, Somalia, where conflict and drought forced populations into makeshift camps. Their findings confirm elevated mortality, particularly among children. Sabah similarly focused on the Gaza Strip's internally displaced populations, documenting severely inadequate housing, water, and electricity. Both pieces urge sustained surveillance and humanitarian interventions.

Several works concentrate on refugees outside their home countries. Al-Rousan et al. reported extremely low medication usage (1.6 in Lebanon and none in Denmark) for hypertension among Syrian refugees in both Lebanon and Denmark, despite high stage 2 hypertension prevalence. Majnoonian et al. identified food insecurity as a pressing issue for Armenians displaced by the 2020 Nagorno-Karabakh conflict, especially for female-headed households and those in collective centers. Kardas et al. tracked healthcare barriers for Ukrainian refugees scattered across Europe, noting how limited information, waiting times, and costs hamper continuity of care—particularly for chronic conditions.

In a complementary qualitative study focused on Lithuania, Urbanavičė et al. explored the lived experiences of Ukrainian refugees, emphasizing barriers to healthcare and social services, especially among Russian-speaking women. Participants reported long waiting times, inadequate psychological support, inconsistent service quality in rural areas, and language difficulties that impeded both access to care and broader integration. The study underscores the importance of improving language support, coordination between services, and providing culturally competent psychological care.

Haight et al. present a community case study describing the establishment of a principles-based community health center in Edmonton, Canada, exclusively serving newcomers. This model emphasizes cultural safety, wraparound psychosocial services, and language support—reflecting some of the solutions lacking in the preceding examples, including those identified by Urbanavičė et al.

### 2.3 Disease burdens

Zhang et al. used the Global Burden of Disease 2021 dataset to demonstrate that while amputation incidence and prevalence among youth (0–19 years) declined globally over three decades, conflict-affected countries (like Syria and Afghanistan) experienced increases. The authors call for improved trauma care and rehabilitative support.

War also undermines preventive measures. Ciccacci et al. trace how armed conflict severely disrupts vaccination campaigns, fuelling polio and measles outbreaks in Syria, Nigeria, Afghanistan, and beyond. They illustrate the importance of “immunization ceasefires” and mobile teams to maintain coverage in war-torn areas. Meanwhile, in Tigray, Ethiopia, Gebru et al. show how overlapping crises—war plus COVID-19—aggravate inequities and stall disease control.

### 2.4 Maternal and reproductive health

Within war-affected Ethiopia, Kebede et al. report increased rates of severe maternal outcomes and maternal near-miss at a referral hospital in the Amhara Region, attributing these patterns to delays and dysfunctions along the obstetric emergency continuum—transport bottlenecks, disrupted referral pathways, and constrained resource availability. Consonant structural signals emerge elsewhere in this Research Topic: in Gaza, as reported by Alamrain et al. the extreme trauma load compelled the Orthopedic Department at Al-Aqsa Martyrs' Hospital to repurpose obstetric/gynecological theaters and delivery rooms for trauma surgery, illustrating how conflict can divert critical capacity away from the childbirth pathway toward trauma care, with predictable repercussions for time-to-intervention and maternal–fetal safety. In parallel, as reported by Ciccacci et al. interruptions to preventive programmes during wartime—well documented for immunization, including cold-chain breakdown, suspended campaigns, and negotiated access—inevitably affect core MCH functions (e.g., tetanus toxoid in pregnancy, neonatal follow-up), amplifying the indirect risk profile for maternal and infant outcomes. Taken together, these strands strengthen the case for conflict-sensitive obstetric referral systems, protected emergency transport, pre-allocation of dedicated delivery theaters and teams, and the maintenance of essential preventive packages (MCH and immunisations) throughout peaks of violence.

### 2.5 Mental health and psychosocial outcomes

Alnaser et al. studied the 2023 Gaza conflict's psychological spillover in Kuwait, identifying moderate GAD-7 scores and significant somatic complaints, suggesting that war-related distress can extend across borders. Likewise, Airapetian et al. measured PHQ-9 depression in Lithuania before and after Russia's invasion of Ukraine, detecting an initial surge in depressive symptoms, which partially subsided over a year. These highlight the cross-border ramifications of war for neighboring populations. Of course these observations are not exempt from methodological problems, being based on a pre-post study design without a concurrent control group. Though inferring causality is arduous, these studies serve as alarm signals that deserve further investigation. The problem of the strength of evidence is addressed by Colombo and Altare. They stress that despite progress in evidence generation and information management, large knowledge gaps remain, and decisions are frequently influenced by political and organizational considerations, rather than by data.

A systematic review by Dönmez et al. underscores complicated grief among adult refugees who have lost family or friends, calling for culturally sensitive diagnostic tools and interventions. The broad psychosocial toll resonates with multiple other studies describing stress among refugees, IDPs, or local communities.

### 2.6 Human rights violations and civilian casualties

Multiple investigations adopt a rights-based perspective. Ayoub et al. quantify shifting mortality ratios between civilians and combatants in Israel-Gaza conflicts, revealing increased targeting of civilians in more recent hostilities. Giacaman et al. introduce an “ecological perspective” instrument for measuring human rights violations in the Israeli-occupied West Bank, highlighting a synergy of oppression from family, community, local authorities, and occupying forces that intensifies the suffering of Palestinians.

## 3 Discussion

While this Research Topic primarily focuses on the consequences of conflict, as experts in public health and epidemiology, we must also critically examine the underlying causes or risk factors. Consequently, our recommendations should not be limited to interventions aimed solely at mitigating the impacts of conflicts but should extend to preventive measures. It is essential to recognize that warfare results not merely from immediate political tensions or power struggles (*proximal causes*), but also from deeper structural factors and complex “risk factors” that precede and facilitate conflicts. Examples include the diffusion of militaristic cultures within societies, even those not directly engaged in conflicts, and significant economic interests, particularly those related to the arms industry. The commodification of weapons and their highly technological nature are two characteristics that contribute to decoupling their use from the perception of the consequences, mainly affecting civilians, and therefore of the associated moral responsibility.

The global arms industry has experienced significant growth in recent years, underscoring the substantial economic interests tied to military production and trade. According to the Stockholm International Peace Research Institute (SIPRI), revenues from the sales of arms and military services by the 100 largest companies in the industry reached $632 billion in 2023, marking a real-terms increase of 4.2% compared with 2022. The United States has solidified its position as the world's leading arms exporter, accounting for 43% of global arms exports between 2020 and 2024. In the same period, European arms imports surged by 155% compared to the previous 4 years, with Europe representing 28% of global arms imports, up from 11% (19).

Addressing these root (*distal*) causes is imperative for achieving sustainable peace and protecting population health globally.

The ethical and professional responsibility of public health practitioners extends beyond merely mitigating the harmful effects of war to encompass active engagement in conflict prevention. This duty is clearly articulated in the Ottawa Charter for Health Promotion, which emphasizes the necessity of promoting peace as a fundamental determinant of health. Different approaches to peace, such as military deterrence vs. antimilitarism, have been debated extensively. Evidence increasingly critiques military deterrence as an ineffective strategy with detrimental impacts on global public health. The prolonged conflict in Afghanistan exemplifies how reliance on military solutions has failed to secure lasting peace, instead perpetuating severe public health crises (20–22). Consequently, public health professionals have a critical role in conflict prevention and peacebuilding efforts, and, importantly, this role can be based on scientific evidence. Their research and advocacy uniquely contribute by highlighting the substantial health costs of war, counterbalancing narratives that frequently focus solely on perceived strategic benefits. Direct engagement from the public health community is essential to influence international policy, ensuring that the extensive and enduring health impacts of conflict are adequately recognized and addressed.

## 4 Perspectives for further research

Our Research Topic is necessarily limited and is only a partial expression of public health research on war. It raises questions about multiple gaps in knowledge. First, estimates of victims are uncertain by definition, and the work of reconstruction of what exactly happened—to civilians in particular—in war theaters is extremely difficult. Inquiries need to be encouraged also from a public health perspective, i.e., considering not only the direct consequences but also the indirect ones we have indicated. Second, the methodology to improve estimates and causal inferences needs to be consolidated. By causal inference here we are not talking about a discussion on military responsibilities in specific episodes (which is not within the remit of public health), but a framework to understand to what degree health consequences are attributable to war episodes. This is something like the “World Weather Attribution“ exercise, that attributes probabilistically single extreme events to climate change (23). Similarly, not only proximal events like immediate casualties, but also distal health outcomes can receive a probabilistic assessment of their relationship with single war episodes and with war more broadly. Third, such a reconstruction of causal networks may help prevention efforts. It is important to be able to predict (e.g., by modeling) the extent of a humanitarian crisis, including infectious diseases, famine, collapse of health systems, etc., having preparedness in mind. In fact, analogies with preparedness to climate change and to epidemics can be illuminating [see for example a list of all the potential aspects involved in preparedness to pandemics reported by *Accademia Nazionale dei Lincei*, many of them are in common with the aftermath of war (24)].
