# Correction: Editorial: Global health and warfare: assessing the broad impacts of conflict on public health

**DOI:** 10.3389/fpubh.2025.1720180

**Published:** 2025-10-16

**Authors:** Stefano Orlando, Paolo Vineis, Pirous Fateh-Moghadam

**Affiliations:** ^1^Department of Biomedicine and Prevention, Università degli Studi di Roma Tor Vergata, Roma, Italy; ^2^School of Public Health, Imperial College London, London, United Kingdom; ^3^Department of Prevention, Local Health Unit, Trento, Italy

**Keywords:** armed conflict, refugees and IDPs, maternal and child health (MCH), mental health, immunization, human rights, humanitarian health response, global health

A correction has been made to the section Introduction, 1^st^ paragraph.

In the published article, there was a mistake in the Introduction. The following sentence read “According to the World Health Organization, ~90% of conflict-related casualties since World War II have been among civilians, highlighting the disproportionate toll that modern conflicts exact on noncombatants (1).” The correct sentence is “According to the World Health Organization, ~60% of conflict-related casualties since World War II have been among civilians, highlighting the disproportionate toll that modern conflicts exact on noncombatants (1).”

The original version of this article has been updated.

